# Bisphosphonates for Secondary Prevention of Osteoporotic Fractures: A Bayesian Network Meta-Analysis of Randomized Controlled Trials

**DOI:** 10.1155/2019/2594149

**Published:** 2019-11-19

**Authors:** Lei Shi, Nan Min, Fei Wang, Qing-Yun Xue

**Affiliations:** Department of Orthopeadics, Beijing Hospital, National Center of Gerontology, 100730 Beijing, China

## Abstract

**Purpose:**

To investigate the comparative efficacies of the five most commonly used bisphosphonates for the secondary prevention of osteoporotic fractures in a Bayesian network meta-analysis.

**Methods:**

Five databases and the reference lists of all acquired articles from inception to July 2017 were searched. A Bayesian random-effects model was employed, and vertebral, hip and nonvertebral nonhip fractures were assessed by odds ratios (ORs) and 95%credible intervals. Furthermore, with respect to each endpoint, rank probabilities for each bisphosphonate were evaluated using the surface under the cumulative ranking curve (SUCRA) value.

**Results:**

Thirteen eligible studies were identified involving 11,822 patients with osteoporotic fractures. Overall in the pairwise meta-analyses, bisphosphonate use significantly reduced the risk of new vertebral, hip, and nonvertebral nonhip fractures, with ORs and 95% confidence intervals of 0.56 (0.49–0.64), 0.69 (0.48–0.98), and 0.82 (0.70–0.97), respectively. In network meta-analyses, significant differences were found between placebo and any one of the five bisphosphonates for new vertebral fractures. The rank probability plot and the SUCRA calculation results suggested that alendronate was the best intervention (14.6%) for secondary prevention of vertebral fractures, followed by zoledronate (15.3%) and etidronate (22.1%). In terms of the incidence of new hip fractures, alendronate was associated with the lowest incidence (18.5%), followed by zoledronate (43.1%) and risedronate (52.5%). However, zoledronate ranked lowest (16.6%) regarding the incidence of new nonvertebral nonhip fractures, followed by risedronate (23.8%) and alendronate (44.1%).

**Conclusions:**

Bisphosphonates show significant efficacy for secondary prevention of new vertebral fractures, and alendronate is most likely to be successful at secondary prevention of vertebral and hip fractures compared with the other four bisphosphonates.

## 1. Introduction

Osteoporosis, which primarily affects postmenopausal women and the elderly population, is characterized by low bone mineral density and decreased bone strength and is the leading cause of fragility fractures, namely, osteoporotic fractures, including vertebral, hip, and nonhip nonvertebral fractures [1, 2]. Osteoporotic fractures result in health and life quality deterioration, which in turn creates a heavy burden for patients and health system. Beyond the age of approximately 50 years, 22% of men, and 50% of women will experience an osteoporotic fracture. As a consequence, these patients are at an increased risk of several adverse outcomes, such as subsequent fracture, morbidity, and mortality [3, 4].

Hence, prevention of osteoporotic fractures is the main therapeutic target in osteoporosis treatment, and medication is a crucial approach, with bisphosphonates being the most commonly prescribed modality [5]. In the United Kingdom, about 10% of females aged 70 years or older with osteoporosis are prescribed bisphosphonates, making it one of the most frequently prescribed drug class in this patient population [6–8].

A large number of randomized controlled trials (RCTs) have demonstrated the effectiveness of different bisphosphonates for fracture prevention [9–16], but little is known about the comparative efficacies of different bisphosphonates for the prevention of secondary fractures. A network meta-analysis can be used to integrate all RCTs that compare different bisphosphonates directly or with placebo while fully preserving randomization [17].

Therefore, we aimed to assess the efficacies of the five most commonly used bisphosphonates (alendronate, ibandronate, risedronate, zoledronate, and etidronate) for the secondary prevention of osteoporotic fractures via an integrated analysis of all available direct and indirect evidence in a Bayesian network meta-analysis.

## 2. Materials and Methods

### 2.1. Search Strategy

Relevant studies published from database inception to July, 2017 were retrieved from the Cochrane Central Register of Controlled Trials (CENTRAL), PubMed MEDLINE, Embase, Chinese National Knowledge Infrastructure (CNKI), and Chinese Wanfang Data Knowledge Service Platform. The keywords used in the searches were: osteoporotic fractures, bisphosphonates (alendronate, ibandronate, risedronate zoledronate, and etidronate), and secondary prevention. We searched PubMed MEDLINE with the use of the combination of medical subject headings (MeSH) and keywords. Two reviewers (LS and FW) independently conducted the initial search through the step of screening all retrieved titles and abstracts. Irrelevant reports were excluded, while the full text of the studies included for eligibility was reviewed. We also manually checked the reference lists of all acquired articles for additional relevant studies.

### 2.2. Inclusion and Exclusion Criteria

The academic studies identified for this network meta-analysis had to meet the following criteria: (1) designed as a RCT; (2) included postmenopausal women or men over 50 years with existing osteoporotic fractures; (3) included a comparison between at least one of the five bisphosphonates, including alendronate, ibandronate, risedronate, zoledronate, and etidronate, with placebo or another of the investigated bisphosphonates; (4) reported clinical outcomes including new vertebral fractures, new hip fractures, or new nonvertebral nonhip fractures, with new vertebral fractures defined as the primary outcome, and new nonvertebral fractures defined as a secondary outcome; (5) provided sufficient and qualified data that could be extracted from original academic studies; and (5) had a treatment duration of at least 24 months.

Studies were excluded if: (1) the patients did not have osteoporotic fractures; (2) the study was not a RCT or a conference abstract or paper, case report, observational study, reviews or duplicated paper; (3) sufficient and qualified data were unavailable; (4) the treatment duration was less than 24 months; and (5) included patients with secondary osteoporosis (glucocorticoid-induced osteoporosis, etc.).

### 2.3. Data Extraction and Quality Assessment

Two reviewers (LS and NM) independently extracted the data from the included academic studies using a standardized data collection form. Discrepancies between the two reviewers were settled by discussion with a third reviewer (QYX) to reach agreement. The authors of the relevant studies were also contacted if additional information was required. New vertebral fracture was chosen as the primary outcome, since it is the most frequently encountered osteoporotic fracture [2, 18], while new hip fractures and nonvertebral nonhip fractures were secondary endpoints. Detailed information from the original articles was extracted, including the study design, name of the first author, year of publication, sample size of enrolled patients, mean patient age, intervention, preparations and doses, treatment cycle, duration, and clinical outcomes (new vertebral fractures, new hip fractures, or new nonvertebral nonhip fractures). The methodological quality of the eligible articles was assessed using the risk of bias as detailed in the Cochrane Collaboration Handbook.

### 2.4. Statistical Analysis

A pair-wise meta-analysis combining studies addressing the same clinical outcome was performed using STATA 14 (Stata Corp, College Station, TX), and clinical outcomes were assessed using odds ratios (ORs) with 95% confidential intervals (CIs). Significant heterogeneity was expressed by *I*^2^ > 50%, which was calculated using the DerSimonian and Laird method with a random effects model; otherwise, the Mantel-Haenszel method with a fixed-effects model was used.

Except for pair-wise meta-analyses, a network meta-analysis for indirect treatment comparison was conducted within a Bayesian framework with a random-effects model [19], which enabled specific incorporation of multiple treatments constructed from two studies that have one of the five bisphosphonates in common and combined indirect and direct evidence for any provided pair of bisphosphonates and certain clinical results. ORs with 95% credible intervals (CrIs), calculated by the Markov chain Monte Carlo method, were obtained using WinBUGS (MRC Bio-statistics Unit, Cambridge, UK). Then, we performed a sensitivity analysis to verify the robustness of the clinical outcomes.

In addition, the consistency between indirect and direct comparisons was assessed through the comparison of ORs from the pair-wise meta-analyses and the pooled ORs from the network meta-analyses. The node-splitting method, calculating the inconsistency of the model for evaluating the consistency, was performed using the software program R (version 3.4.0), in which the Bayesian *P* value is considered as the inconsistency [20]. Based on bisphosphonates' rank probabilities, we sorted the included bisphosphonates according to each clinical outcome. The sum of the rank probabilities for each bisphosphonate was assessed by the surface under the cumulative ranking curve (SUCRA) [21]. A lower SUCRA for a given intervention indicates that it is more efficient for the secondary prevention of osteoporotic fractures. Additionally, sensitivity analyses were performed to evaluate the influence of each study on the overall results.

## 3. Results

### 3.1. Study Characteristics

**Figure 1:**
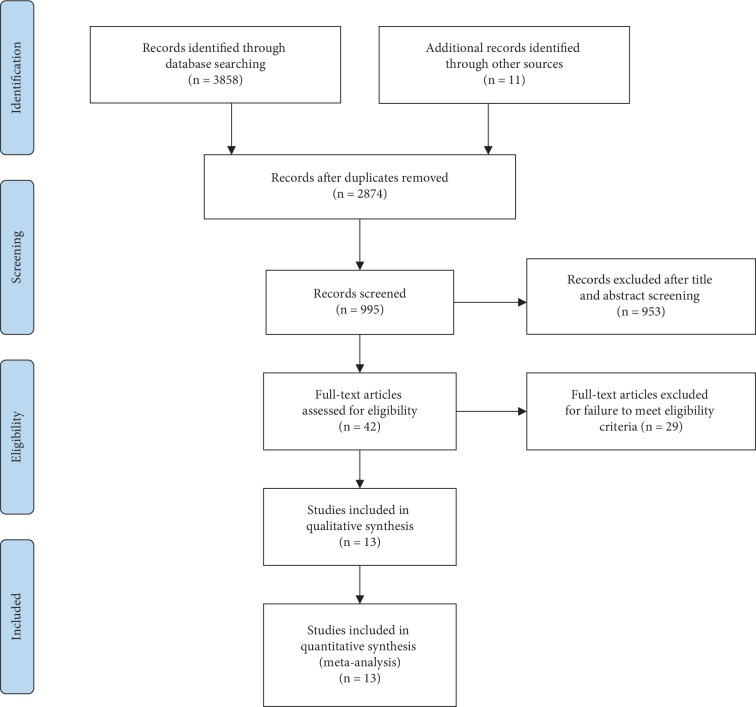
Flow diagram of review process.

Of 3869 records that were initially identified from the literature search, 13 academic papers remained after removal of duplicates and screening by scanning titles, abstracts, and full texts, with a total of 11,822 patients with existing vertebral fractures[13–16, 22–30], of which postmenopausal women accounted for more than 98%. A flow chart of study selection is presented in Figure 1. The patients enrolled received pharmacotherapy using alendronate, ibandronate, risedronate, zoledronate, or etidronate. The baseline characteristics and primary outcomes of eligible studies were categorized by the bisphosphonates used and are summarized in Table 1. The robustness of the results of pair-wise meta-analysis was further verified through a sensitivity analysis and funnel plot as shown in Figures [Supplementary-material supplementary-material-1] with credible results and no obvious publication bias.

The quality of the included trials was moderate to high, as shown in Table 1, with 61.5% of the studied papers considered as having a low risk of bias for blinding of outcome assessors [14, 16, 23, 24, 26–29], 100% for blinding of patients [13–16, 22–30], and 38.5% for incomplete outcome data [15, 22, 27, 28, 30]. None of the studied papers was judged to have a high risk of bias for any item of the methodological quality evaluated, except for allocation concealment, for which 12 (92.3%) of the 13 studied papers were judged as an unclear risk of bias [13–16, 22–25, 27–30].

### 3.2. Pairwise Meta-Analysis

Compared with placebo, bisphosphonates significantly reduced the risk of new vertebral, hip, and nonvertebral nonhip fractures, with ORs and 95% CIs of 0.56 (0.49–0.64), 0.69 (0.48–0.98), and 0.82 (0.70–0.97), respectively ([Supplementary-material supplementary-material-1]).

### 3.3. Network Meta-Analysis

**Figure 2:**
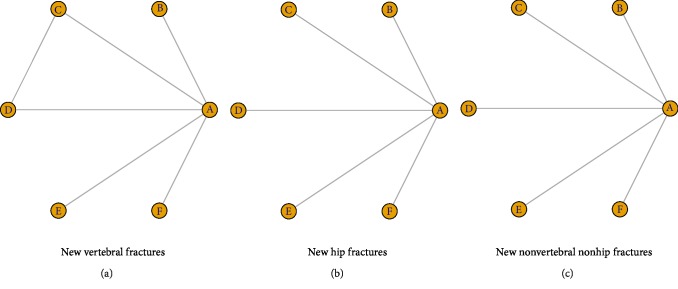
Evidence network of eligible comparisons for Bayesian network meta-analysis according to (a) new vertebral fractures, (b) new hip fractures, and (c) new nonvertebral nonhip fractures (A, Placebo; B, Alendronate; C, Ibandronate; D, Risedronate; E, Zoledronate; F, Etidronate).

**Figure 3:**
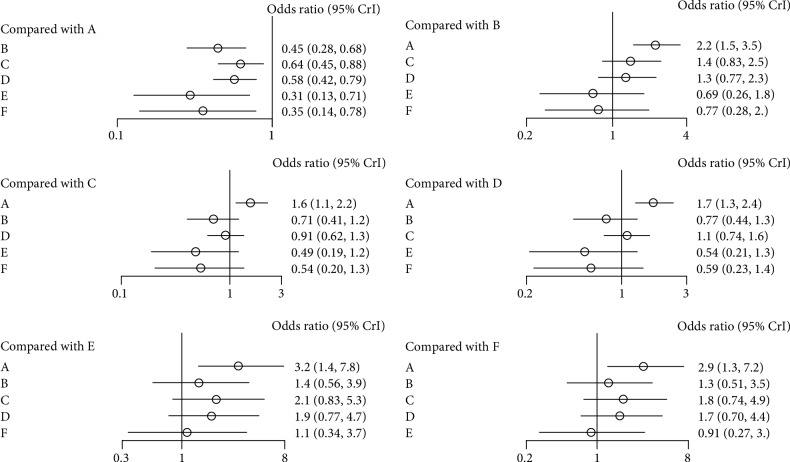
Forest plot for new vertebral fractures (A, Placebo; B, Alendronate; C, Ibandronate; D, Risedronate; E, Zoledronate; F, Etidronate).

Figure 2 shows the network diagram of eligible studies. As the primary outcome of this Bayesian analysis, the incidence of new vertebral fractures was compared among the treatments (Figure 3). From the 13 papers selected for including direct or indirect comparisons, we found that all five bisphosphonates were more effective than placebo (alendronate: OR = 0.45, 95% CrI 0.28–0.68; ibandronate: OR = 0.64, 95% CrI 0.45–0.88; risedronate: OR = 0.58, 95% CrI 0.42–0.79; zoledronate: OR = 0.31, 95% CrI 0.13–0.71; and etidronate: OR = 0.35, 95% CrI 0.14–0.78). Zoledronate had the lowest OR, but no significant difference was found in comparison with any other bisphosphonate.

**Figure 4:**
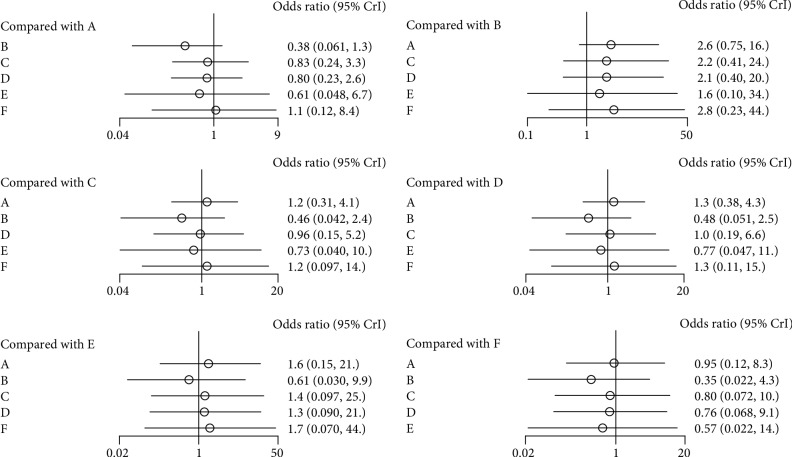
Forest plot for new hip fractures (A, Placebo; B, Alendronate; C, Ibandronate; D, Risedronate; E, Zoledronate; F, Etidronate).

Nine studies were included in the analysis concerning the efficacy for secondary prevention of new hip fractures. Alendronate had the lowest OR at 0.38, followed by zoledronate and risedronate, and the OR for etidronate surpassed 1. However, none of the five bisphosphonates exhibited a statistically significant superiority to placebo (Figure 4). Moreover, comparison among the five studied bisphosphonates did not reveal any statistically significant difference either.

**Figure 5:**
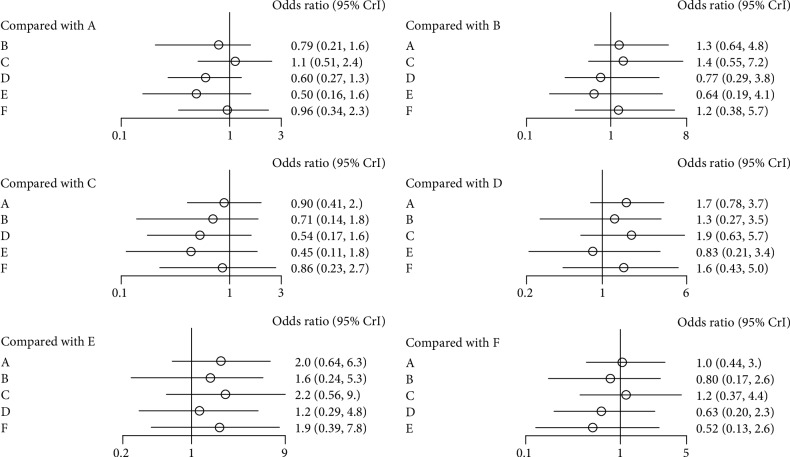
Forest plot for new nonvertebral nonhip fractures (A, Placebo; B, Alendronate; C, Ibandronate; D, Risedronate; E, Zoledronate; F, Etidronate).

Nine studies investigated the effectiveness of bisphosphonates for the secondary prevention of new nonvertebral nonhip fractures. A Forest plot for new nonvertebral nonhip fractures is shown in Figure 5. According to our results, the efficacies of bisphosphonates were similar to those of placebo (alendronate: OR = 0.79, 95% CrI0.21–1.6; ibandronate: OR = 1.1, 95% CrI0.51–2.4; risedronate: OR = 0.6, 95% CrI 0.27–1.3; zoledronate: OR = 0.50, 95% CrI 0.16–1.6; etidronate: OR = 0.96, 95% CrI 0.34–2.3). No statistically significant difference was found in a comparison among the five studied bisphosphonates.

### 3.4. Comparisons between Direct and Indirect Evidence

**Figure 6:**
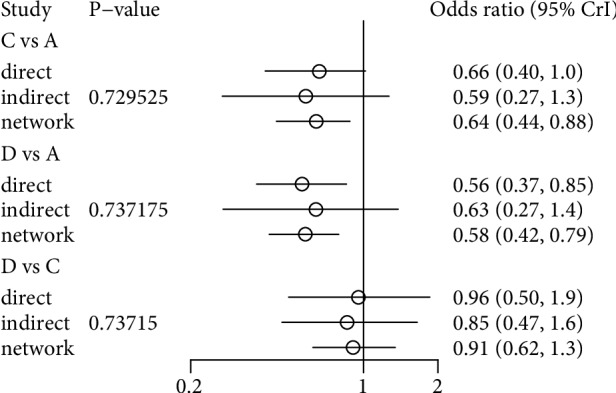
Comparison of direct and indirect evidence (A, Placebo; B, Alendronate; C, Ibandronate; D, Risedronate; E, Zoledronate; F, Etidronate).

The node-splitting method comparing indirect and direct evidence for a specific comparison of bisphosphonates and its Bayesian *P* value were used to demonstrate the inconsistency between the direct and indirect comparisons in our results. The general consistency from direct and indirect evidence was identified in the comparison of ibandronate and risedronate for secondary prevention of vertebral fracture with corresponding *P* values of 0.730, 0.737, and 0.737, respectively, with no significant inconsistency found (Figure 6).

### 3.5. Relative Ranking of Five Interventions

SUCRAs were applied to provide a probability rank for each bisphosphonate. The results for the five bisphosphonates are shown in Table 2. As mentioned above, the lower the SUCRA of an active intervention, the more efficient it is, indicating a lower incidence of secondary osteoporotic fractures. With respect to the primary endpoint of new vertebral fractures, alendronate was the best treatment based on its lowest probability ranking (14.6%), followed by zoledronate (15.3%) and etidronate (22.1%). In terms of new hip fractures, alendronate ranked lowest (18.5%), followed by zoledronate (43.1%), and risedronate (52.5%). However, zoledronate ranked lowest (16.6%) regarding the incidence of new nonvertebral nonhip fractures, followed by risedronate (23.8%) and alendronate (44.1%).

## 4. Discussion

Our meta-analysis demonstrated that bisphosphonates significantly reduced the risk of secondary new vertebral, hip, and nonvertebral nonhip fractures. Alendronate was identified as the most efficacious for secondary prevention of vertebral and hip fractures by probability plot and SUCRA calculation, while zoledronate showed better performance for nonvertebral nonhip fracture prevention. However, for all fracture endpoints combined, no significant difference was found among the five bisphosphonates. To our knowledge, this is the first Bayesian network meta-analysis to compare the efficacies of the five most commonly used bisphosphonates for the secondary prevention of osteoporotic fractures. The results could be used as an important reference for decision making in clinical scenarios.

The goal of osteoporosis management is to prevent osteoporotic fractures, but for those who have had sustained an osteoporotic fracture, it is more urgent to prevent a secondary fracture. This is because patients with an osteoporotic fracture are more likely to experience a recurrent fracture, with a marked increase of morbidity and mortality compared to those among patients without fractures [31–35]. For patients with hip fractures, the estimated 1-and 2-year morality rates in South Korea were reported to be 16% and 28%, respectively, and the prevalence of osteoporotic fractures as well as the associated societal costs are estimated to increase markedly given that the aging index is expected to increase up to 213.8% by 2030 [36]. In mainland China, the pooled estimate for the 1-year mortality rate following hip fracture was 13.96% between the years 2000 and 2018 [37]. Also, vertebral fractures occurred with a high prevalence in the very elderly population, with an estimated incidence of 30.4% according to the vertebral fracture assessment [38]. Bisphosphonates are well-studied antiresorptive medications that are widely approved and recommended as a first line choice for osteoporosis in postmenopausal women and older populations. Several high-quality RCTs have demonstrated the efficacy of individual bisphosphonates for secondary fracture prevention, but few sufficient comparisons have been carried out due to a lack of large-scale direct trials.

Currently available meta-analyses and reviews have largely focused on anti-osteoporosis medication for primary fracture prevention [9–11]. A network meta-analysis reported that teriparatide, bisphosphonates, and denosumab are most effective at reducing the risk of fragility fractures, even though the differences in efficacy across the studied interventions were small [39]. A similar Bayesian network meta-analysis that compared 10 therapies (the five bisphosphonates in our study along with clodronate, raloxifene, parathyroid, hormone, denosumab, and strontium ranelate) was initiated by Wang et al. [5], and they suggested that zoledronate and parathyroid hormone have the highest probability of providing the best overall osteoporotic fracture protection with satisfactory performance. However, conflict also exists with some comparisons. As reported by Sanderson et al., in their study of the relative effect of bisphosphonates (alendronate, ibandronate, risedronate, and zoledronate), no active intervention was considered to be more effective than any other one for preventing fracture [40].

In a systematic review by the Cochrane library about “alendronate in secondary prevention of osteoporotic fractures” [41], which indicated that both clinically important and statistically significant reductions in vertebral, nonvertebral, hip, and wrist fractures were observed with alendronate for secondary prevention of fracture, the definition of secondary prevention was “women whose bone density was at least 2 SD values below the peak bone mass or who had experienced previous vertebral compression fractures”. In another systematic review and meta-analysis by Saito et al. that included patients without osteoporotic fractures, secondary fragility fractures were prevented by several anti-osteoporotic drugs, among which bisphosphonates and PTH were most effective at preventing nonvertebral fractures [42]. However, this is not exactly in accordance with the strictly defined secondary prevention of osteoporotic fractures as in the Fracture Liaison Service (FLS) project initiated by International Osteoporosis Foundation (IOF) [43–45] and clinical practice, which is the target population we would like to investigate in our study.

Therefore, previous studies enrolled patients both with and without existing osteoporotic fractures, and conclusions about different interventions for secondary fracture prevention cannot be inferred from findings in patients with varying fracture risks.

Moreover, since the comparative efficacies of bisphosphonates for secondary fracture prevention are hard to assess by direct comparison through clinical trials, the possible ranking applied in this network meta-analysis may provide a valuable prediction. Alendronate showed better performance in our study for secondary prevention of vertebral and hip fractures, while zoledronate was recommended for nonvertebral nonhip fracture prevention, which is partially in accordance with a network meta-analysis conducted by Jansen et al. in primary prevention [46]. Jansen et al.'s study suggested that alendronate, ibandronate, risedronate, and zoledronate are all effective for the prevention vertebral fracture with zoledronate as a better choice and alendronate ranked first for preventing hip fracture. The discrepancy in efficacy might be due to differences in the inclusion criteria and the baseline characteristics of the study population.

Both men and postmenopausal women with osteoporotic fractures were enrolled in the analysis, which included two alendronate trials, one ibandronate trial, and one zoledronate trial with 148 men altogether. In addition, most existing fractures are prevalent vertebral fractures. In our preliminary literature search, one article about zoledronate use in patients with a previous hip fracture was identified, but it was then excluded because the median treatment duration was less than 2 years. In addition, in the analysis of alendronate, different doses were given in the included trials (from 5 mg/d to 10 mg/d or 70 mg/w) which might underestimate the efficacy in fracture prevention.

There are some limitations in the present meta-analysis. First, the diagnostic criterion of new vertebral fracture has not been uniform across different studies, given that both morphometric and clinical vertebral fractures are applied in fracture detection, which might lead to potential bias. Secondly, a relatively small number of new nonvertebral nonhip fractures was investigated in relevant studies, which lacked some key comparisons. Consequently, the results for this endpoint should be interpreted with caution. Third, the baseline characteristics and the compliance varied among different studies, which may influence the detection of the investigated events, and trial durations also differed, possibly adding heterogeneity or bias to the results. Lastly, the methodological quality was somewhat limited because whether allocation concealment was conducted properly was unclear in 12 studies[13–16, 22–25, 27–30]. Also, a high risk of incomplete outcome data bias was observed because the method of last‐observation was carried out for the missing data in some studies [13, 14, 16, 23, 24, 26, 29].

## 5. Conclusions

By combining all the direct and indirect evidence, our results suggest that bisphosphonates showed significant efficacy for secondary prevention of new vertebral fractures, while alendronate had the highest probability of successful performance in the secondary prevention of vertebral and hip fractures compared with the other four bisphosphonates that treat patients with existing osteoporotic fractures. However, more prospective, direct studies with large sample sizes, high quality, and longer follow-up periods are needed to confirm the results of our study.

## Figures and Tables

**Table 1:** Baseline characteristics of the trials included in the present study.

Study	Design	Mean age, (t1/t2) yrs	Interventions		No (t1/ t2)	Study duration	Outcome^∗^	Risk of bias		
			t1	t2				Allocation concealment	Blinding (patients/outcome assessors)	Incomplete outcome data
Nakamura et al., 2016	RCT	74/74	Zoledronate (5 mg/yr )	Placebo	301/296	2 yrs	1, 2, 3	Unclear	Low/Low	High
Wang et al., 2016	RCT	67.3/68.5	Alendronate (70 mg/w 2 yrs)	Placebo	37/37	2 yrs	1	Unclear	Low/Unclear	Low
Ji et al., 2013	RCT	67.3/65.5	Alendronate (70 mg/w 2 yrs)	Placebo	40/40	2 yrs	1, 2, 3	Unclear	Low/Unclear	Low
Nakamura et al., 2013	RCT	72.2/72.9/73	Ibandronate (1 mg/m) vs (0.5 mg/m)	Risedronate (2.5 mg/d)	382/376/376	3 yrs	1	Unclear	Low/Low	High
Recker et al., 2004	RCT	67/67	Ibandronate (1 mg/3 ms) vs (0.5 mg/3 ms)	Placebo	1912/975	3 yrs	1, 2, 3	Unclear	Low/Low	High
Chesnut et al., 2004	RCT	69/69	Ibandronate (2.5 mg/d) vs (20 mg/qod intermittent)	Placebo	977/975	3 yrs	1, 2, 3	Unclear	Low/Low	Low
Kushida et al., 2004	RCT	71.2/72.6	Alendronate (5 mg/d 3 yrs)	Placebo	90/80	3 yrs	1	Unclear	Low/Low	Low
Reginster et al., 2000	RCT	71/71	Risedronate (5 mg/d)	Placebo	408/408	3 yrs	1, 2, 3	Unclear	Low/Unclear	High
Harris et al., 1999	RCT	66/66	Risedronate (5 mg/d)	Placebo	821/820	3 yrs	1, 2, 3	Low	Low/Low	High
Clemmesen et al., 1997	RCT	67/68/70	Risedronate (2.5 mg/d) vs (2.5 mg/d, cyclic)	Placebo	44/44	3 yrs	1	Unclear	Low/Unclear	Unclear
Black et al., 1996	RCT	71/71	Alendronate (5 mg/d 2 yrs, 10 mg/d 1 yr)	Placebo	1022/1005	3 yrs	1, 2, 3	Unclear	Low/Low	High
Watts et al., 1990	RCT	64.7/65.7	Etidronate (400 mg/d, intermittent)	Placebo	105/104	2 yrs	1,2,3	Unclear	Low/Low	High
Storm et al., 1990	RCT	68.3/68.3	Etidronate (400 mg/d, intermittent)	Placebo	33/33	150 weeks	1, 2, 3	Unclear	Low/Unclear	Low

RCT: Randomized controlled trials; No: patients' number; t1: treatment group; t2: control group; yrs: Years; d: daily; w: week; m: month; qod: every other day.

^∗^Outcome (1) New vertebral fractures out of total subjects; (2) New hip fractures out of total subjects; (3) New nonvertebral nonhip fractures out of total subjects.

**Table 2:** Relative ranking of five bisphosphonates according to SUCRA values.

Drugs	New vertebral fractures	New hip fractures	New nonvertebral nonhip fractures
Placebo	0.997	0.701	0.711
Alendronate	0.146	0.185	0.441
Ibandronate	0.701	0.553	0.779
Risedronate	0.580	0.525	0.238
Zoledronate	0.153	0.431	0.166
Etidronate	0.221	0.609	0.642

## Data Availability

The datasets generated and analyzed during the present study are available from the corresponding author on reasonable request.

## Supplementary Materials

Supplementary Materials Figure S1. Pairwise meta-analysis of bisphosphonates versus placebo ((a) new vertebral fractures, (b) new hip fractures, and (c) new non-vertebral non-hip fractures). Figure S2. Sensitivity analysis showed ((a) new vertebral fractures, (b) new hip fractures, and (c) new nonvertebral nonhip fractures). Figure S3. Publication bias ((a) new vertebral fractures, (b) new hip fractures, and (c) new nonvertebral nonhip fractures).
