# Supplementary material for: Bisphosphonates for Secondary Prevention of Osteoporotic Fractures: A Bayesian Network Meta-Analysis of Randomized Controlled Trials
Source: Biomed Res Int. 2019 Nov 19;2019:2594149. doi: 10.1155/2019/2594149 (PMC6885847; doi:10.1155/2019/2594149)
Supplement: Supplementary Materials — Figure S1. Pairwise meta-analysis of bisphosphonates versus placebo ((a) new vertebral fractures, (b) new hip fractures, and (c) new non-vertebral non-hip fractures). Figure S2. Sensitivity analysis showed ((a) new vertebral fractures, (b) new hip fractures, and (c) new nonvertebral nonhip fractures). Figure S3. Publication bias ((a) new vertebral fractures, (b) new hip fractures, and (c) new nonvertebral nonhip fractures). [file 2594149.f1.doc]

Bisphosphonates for secondary prevention of osteoporotic fractures: a Bayesian network meta-analysis of randomized controlled trials

**Supplementary Figure Legends**

**Figure S1.** Pairwise meta-analysis of bisphosphonates versus placebo ((a) new vertebral fractures, (b) new hip fractures, and (c) new non-vertebral non-hip fractures).

**Figure S2.** Sensitivity analysis showed ((a) new vertebral fractures, (b) new hip fractures, and (c) new non-vertebral non-hip fractures).

**Figure S3.** Publication bias ((a) new vertebral fractures, (b) new hip fractures, and (c) new non-vertebral non-hip fractures)


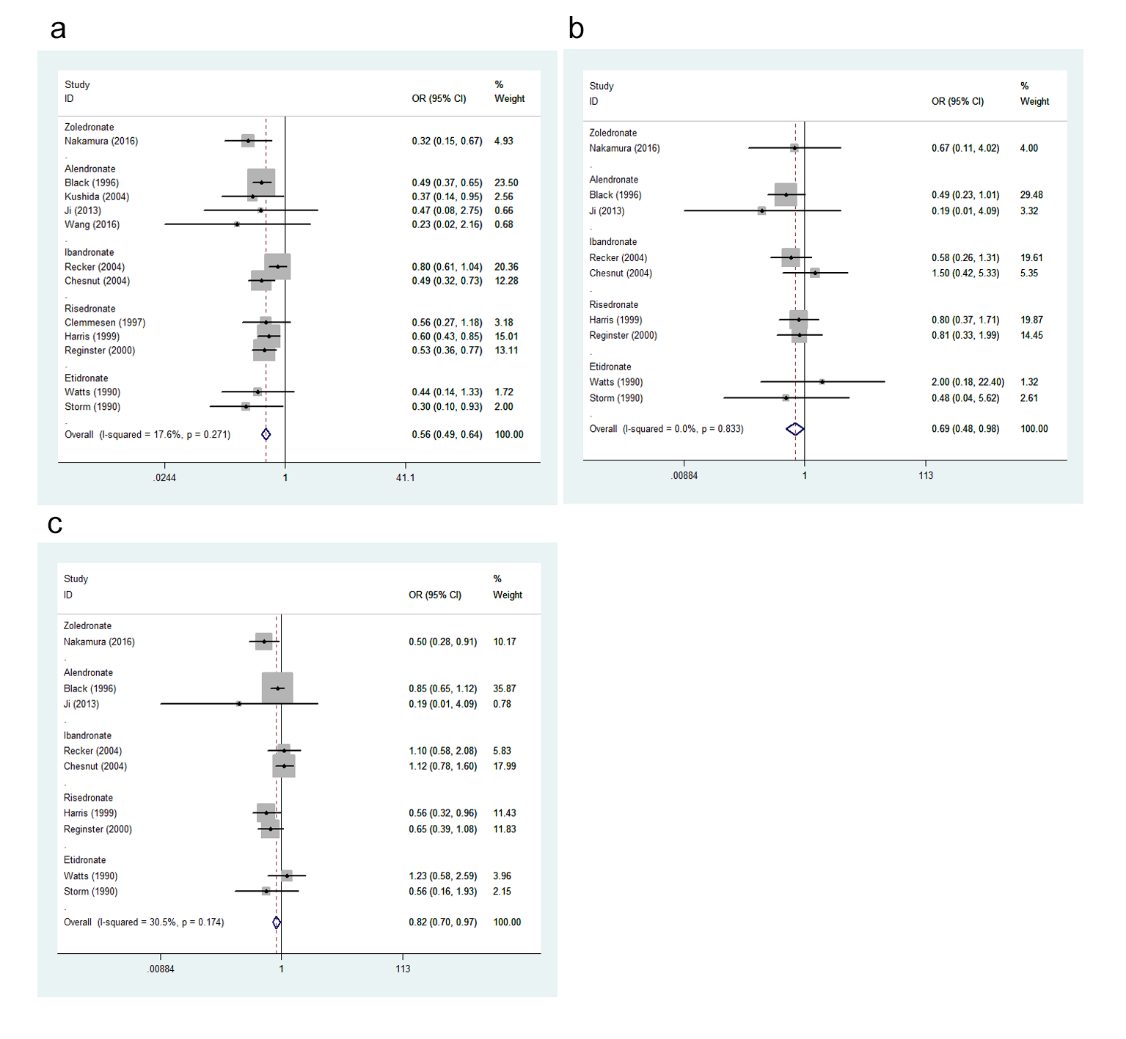


**Figure S1**


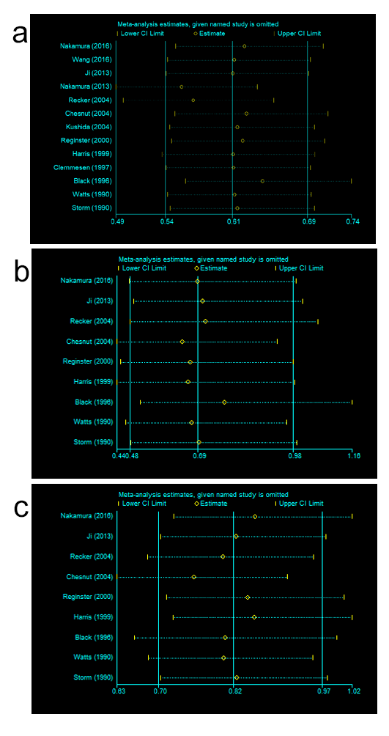


**Figure S2**


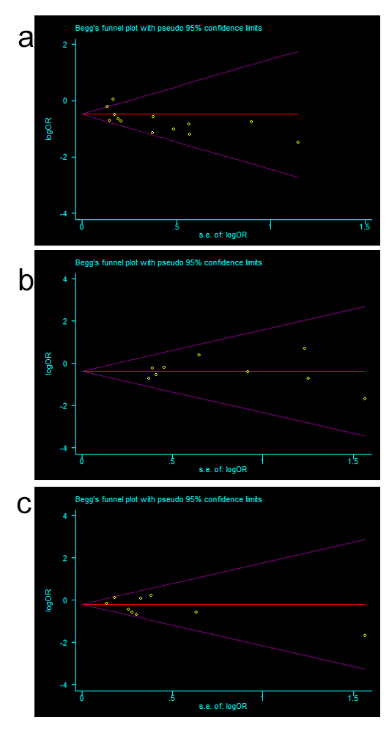


**Figure S3**
